# Transcriptome analysis revealed gene expression feminization of testis after exogenous tetrodotoxin administration in pufferfish *Takifugu flavidus*

**DOI:** 10.1186/s12864-022-08787-z

**Published:** 2022-08-03

**Authors:** Xue He, Hexing Wu, Yaping Ye, Xiaolin Gong, Baolong Bao

**Affiliations:** Key Laboratory of Exploration and Utilization of Aquatic Genetic Resources (Shanghai Ocean University), Ministry of Education; International Research Center for Marine Biosciences at Shanghai Ocean University, Ministry of Science and Technology; National Demonstration Center for Experimental Fisheries Science Education, Shanghai Ocean University, Shanghai, 201306 China

**Keywords:** Tetrodotoxin (TTX), Piwi-interacting RNAs, Transcriptome, Sexual dimorphism, Feminization, *Takifugu flavidus*

## Abstract

Tetrodotoxin (TTX) is a deadly neurotoxin and usually accumulates in large amounts in the ovaries but is non-toxic or low toxic in the testis of pufferfish. The molecular mechanism underlying sexual dimorphism accumulation of TTX in ovary and testis, and the relationship between TTX accumulation with sex related genes expression remain largely unknown. The present study investigated the effects of exogenous TTX treatment on *Takifugu flavidus*. The results demonstrated that exogenous TTX administration significantly incresed level of TTX concentration in kidney, cholecyst, skin, liver, heart, muscle, ovary and testis of the treatment group (TG) than that of the control group (CG). Transcriptome sequencing and analysis were performed to study differential expression profiles of mRNA and piRNA after TTX administration of the ovary and testis. The results showed that compared with female control group (FCG) and male control group (MCG), TTX administration resulted in 80 and 23 piRNAs, 126 and 223 genes up and down regulated expression in female TTX-treated group (FTG), meanwhile, 286 and 223 piRNAs, 2 and 443 genes up and down regulated expression in male TTX-treated group (MTG). The female dominant genes *cyp19a1*, *gdf9* and *foxl2* were found to be up-regulated in MTG. The *cyp19a1*, whose corresponding target piRNA *uniq_554482* was identified as down-regulated in the MTG, indicating the gene expression feminization in testis after exogenous TTX administration. The KEGG enrichment analysis revealed that differentially expressed genes (DEGs) and piRNAs (DEpiRNAs) in MTG vs MCG group were more enriched in metabolism pathways, indicating that the testis produced more metabolic pathways in response to exogenous TTX, which might be a reason for the sexual dimorphism of TTX distribution in gonads. In addition, TdT-mediated dUTP-biotin nick end labeling staining showed that significant apoptosis was detected in the MTG testis, and the role of the cell apoptotic pathways was further confirmed. Overall, our research revealed that the response of the ovary and testis to TTX administration was largely different, the ovary is more tolerant whereas the testis is more sensitive to TTX. These data will deepen our understanding on the accumulation of TTX sexual dimorphism in *Takifugu*.

**Supplementary Information:**

The online version contains supplementary material available at 10.1186/s12864-022-08787-z.

## Introduction

Tetrodotoxin (TTX), one of the most potent neurotoxins which is specific to voltage-gated sodium channels (VGSCs) on excitable membranes of muscle and nerve tissues, was long believed to occur exclusively in TTX-bearing pufferfish, mainly the genus *Takifugu* [1, 2]. TTX is produced primarily by marine bacteria and accumulated extremely high in pufferfish through the food chain with tissue-specific and seasonal changes [3–5]. It is suggested that TTX may function as a chemical defense against predators and as a pheromone during spawning [6, 7].

The accumulation of TTX in pufferfish has been studied extensively [1, 2, 8–11]. The distribution of TTX in pufferfish bodies appears to be species-specific [2, 3, 5, 7, 12–14]. In marine species, the liver and ovary generally have the highest toxicity, followed by the intestines and skin. Muscles and/or testis are non-toxic or weakly toxic [12]. From a seasonal perspective, whole body TTX content was significantly higher during the maturation/spawning period than in the ordinary period [3]. Accumulation of TTX also demonstrated sex dimorphism, ovary generally accumulated a higher level of TTX than the testis [2, 3, 5, 7, 12, 13]. Interestingly, through all seasons, TTX was detected in the skin or ovary of females and the skin or liver of males in *Takifugu. poecilonotus* and *Takifugu niphobles* [4, 11, 15], the difference in TTX accumulation between females and males was particularly evident during the spawning period. These tissue-specific seasonal changes in TTX content suggest that TTX accumulation might be affected by sexual maturation [3].

The larvae of *Takifugu* pufferfish were protected by maternal TTX which had accumulated in the eggs during their development in the ovary [16, 17]. In addition, studies have shown that TTX as a toxin or pheromone accumulated in the ovary protects pufferfish larvae from predators [6, 16, 17]. Although TTX accumulation demonstrates sexual dimorphism in gonads of pufferfish, the molecular mechanism underlying and the relationship between TTX accumulation and steroidogenesis, sex differentiation and reproduction-related genes transcriptome analysis remained unclear.

Transcriptome analysis technologies are important systems-biology methods for the investigation of the organisms' stress response, gene expression, and regulatory pathways changes. Transcriptome refers to the set of all RNA molecules from protein-coding (mRNA) to noncoding RNA, including rRNA, tRNA, lncRNA, pri-miRNA, and others [18, 19] Piwi-interacting RNAs (Piwi/piRNAs) are single-stranded, 26–32 nt length small RNAs that function by interacting with Piwi proteins to form RNA–protein complexes [20, 21]. Animal studies revealed that piRNAs are limited expressed in a few tissues, such as tumors [22–24], brain and nervous tissue [23, 25–27], whereas generally abundant in gonads of invertebrates [28–32] and vertebrates [23, 33–38]. These results were also consistent with teleosts, including zebrafish (*Danio rerio*) [30, 39], tilapia (*Oreochromis niloticus*) [40], Japanese flounder (*Paralichthys olivaceus*) [41], tongue soles (*Cynoglossus semilaevis*) [42]. Previous studies have shown that fertility and normal gonadal morphology in the female progeny of *Drosophila* requires maternal piRNAs, but not males [43]. piRNAs derived from the W chromosome are expressed more abundantly in the ovary than in the testis of silkworms [44].

The piRNAs play a role in targeting the transcripts of active TEs (Transcriptional elements) and maintaining the methylation pattern of maternal genomic DNA. It was proved that ovarian and testicular piRNAs are two different classes with different functions, and their expression appears to be regulated by distinct transcription factors in humans [45]. Above all, at least a large number of piRNAs showed sexual dimorphism in the testes and ovaries. In addition, putative piRNAs were found abundant in the gonads of *Takifugu rubripes* [46], whereas the function and connection between TTX and piRNAs in the gonads of pufferfish have not been investigated. To gain insights into sexual dimorphism of TTX accumulation in gonads, steroidogenesis, sex differentiation and reproduction-related genes and small non-coding RNAs were chosen to further investigate the molecular mechanisms.

In the present study, we investigated the expression changes of piRNAs and mRNAs in both ovary and testis of *Takifugu flavidus* on the transcriptomic level after the intramuscular injection with TTX. The differentially expressed piRNAs and genes were identified, function and pathway of these piRNAs and genes were also analyzed. At last, apoptosis detection was performed in the TTX-treated gonads. Through transcriptome analysis, the gene expression pattern's feminization of testis in pufferfish *Takifugu flavidus* after exogenous TTX administration was observed, and these data will deepen our understanding on the accumulation of TTX sexual dimorphism in *Takifugu*.

## Results

### Exogenous TTX administration significantly elevated the tissue TTX level of *Takifugu flavidus*

**Fig. 1:**
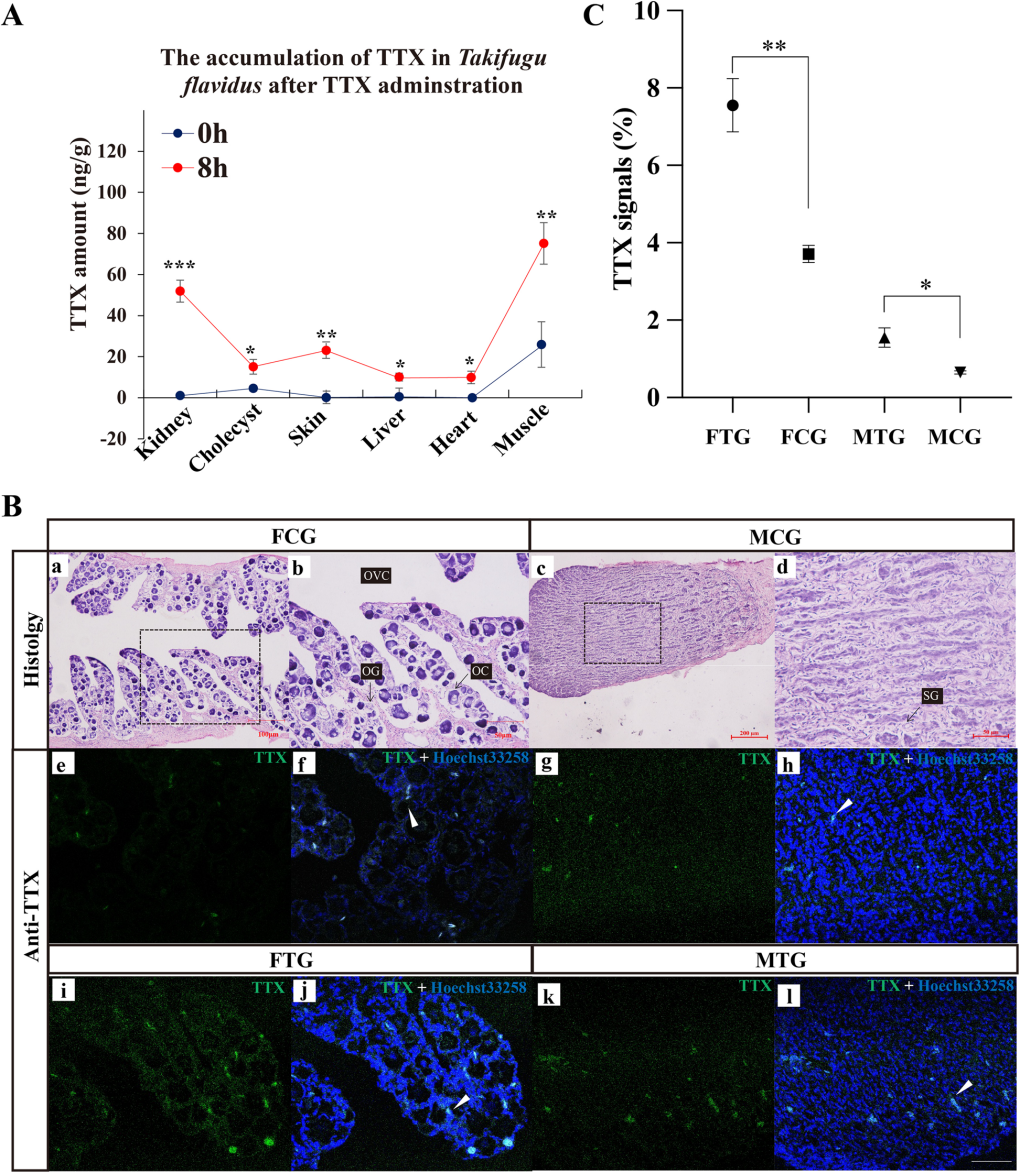
Exogenous TTX administration significantly elevated the tissue TTX level of *Takifugu flavidus*. **A** Different tissues of TTX amount after intramuscular injection of TTX of *Takifugu flavidus*. **B** hematoxylin–eosin staining and TTX immunofluorescence histochemical observation of gonads after intramuscular injection of TTX. b, d, f, h, j, l are the magnifying of a, c, e, g, i, k, respectively. OG, oogonia; OC, oocyte; OVC, ovarian cavity; SG, spermatogonia; The green signals represent TTX and the blue signals represent Hoechst33258 (staining for nucleic acid), the white dotted rectangle indicates the magnified area, black arrows showed the germ cells, the white triangle arrows indicate the positive signals, the scale bar is 100, 200 and 50 μm. C, TTX positive signals statistical analysis of gonads section*.* Data were shown as mean ± SEM. *, indicating that *P* < 0.05; **, indicated that *P* < 0.01

After 8 h of TTX administration, the LC–MS/MS (Liquid Chromatography with tandem mass spectrometry) method was performed to detect the concentration of TTX in different groups and tissues, the results showed that the TTX concentration of TG (the TTX-treated group) is significantly higher than CG (the control group) in kidney, cholecyst, skin, liver, heart, and muscle with mixed-sex (Fig. 1A).

To further explore the accumulation of TTX in the ovary and testis of *Takifugu flavidus*, histological analysis was performed to identify the sex of gonads in 6 months old of *Takifugu flavidus*. Twelve gonads were dissected from each TG and CG. Five ovaries and seven testes were confirmed in the CG, and six ovaries and six testes were confirmed in the TG. All of these gonads are immature, the ovarian cavity, oogonia and oocytes were observed in the ovary (Fig. 1B-a, b). The spermatogonia without spermatocytes or spermatids were observed in the testis (Fig. 1B-c, d). Immunofluorescence histochemical analysis used an anti-TTX antibody demonstrated that TTX was localized at the somatic cells around the oocytes in the FCG (the female control group) and FTG (the female TTX-treated group) ovary with intense signals (Fig. 1B-e, f). Weak and strong TTX signals were detected in the MCG (the male control group) and MTG (the male TTX-treated group) testis, respectively (Fig. 1B-g, h). Algorithms for unified illumination, focus correction, and tiling and stitching enabled us to analyze large fields of cells and quantify the average cellular staining intensity for different TTX, the statistical analysis showed that the total TTX signals in the TG gonads were significantly higher than the CG gonads after TTX administration (Fig. 1C).

### Transcriptome sequencing and novel piRNA identification

Total RNA of gonads (testis or ovary) was isolated and sequenced for piRNA and mRNA analysis from the CG and TG of *Takifugu flavidus*. Overall, 94.91% of FCG, 94.91% of MCG, 98.09% of FTG, and 94.32% of MTG of the clean reads had scored at the Q30 level in mRNA sequencing (Table 1). 85.06% (FCG), 86.10% (MCG), 86.04% (FTG) and 85.63% (MTG) clean reads were matched onto the *Takifugu rubripes* reference genome (*fTakRub1.2*).

**Table 1:** Summary of piRNA and mRNA transcriptome data of *Takifugu flavidus* gonads

Sample group		FCG	FTG	MCG	MTG
piRNA-seq	clean bases	0.983G	0.668G	1.032G	0.841G
	total reads	17,695,205	11,741,016	17,391,118	13,693,446
	uniq reads	2,915,273	2,660,367	2,515,290	3,116,492
	error rate (%)	0.02	0.02	0.02	0.02
	Q20 (%)	99.62	99.64	99.55	99.59
	Q30 (%)	98.86	98.94	98.75	98.79
	GC content (%)	48.9200	49.3300	48.8600	49.1900
	uniq- piRNAs	507,004	472,764	335,106	530,323
	numbers of piRNA (reads ≥ 500)	723	365	403	345
	piRNA clusters	5396	5385	5297	5418
mRNA-seq	raw reads	20,935,814	23,188,308	23,438,065	20,223,260
	clean reads	20,420,252	22,290,909	22,827,658	19,650,444
	total mapped	34,740,218 (85.06%)	38,356,040 (86.04%)	39,307,194 (86.10%)	33,651,476 (85.63%)
	clean bases	6.13G	6.69G	6.85G	5.90G
	error rate (%)	0.02	0.02	0.02	0.02
	Q20 (%)	98.28	98.2	98.04	98.09
	Q30 (%)	94.91	94.91	94.32	94.62
	GC content (%)	52.31	53.58	50.89	52.91

*FCG* Female control group, *FTG* Female TTX-treated group, *MCG* Male control group, *MTG* Male TTX-treated group

piRNA sequencing results showed that the peak value of all groups was mainly concentrated at 28 bp (Figure S1A), with the character of 1U-bias of the molecule (Figure S1B), which was consistent with the characteristic length and structure bias of piRNAs. The numbers of unique/novel piRNAs for the four groups were calculated, there were 32,089, 20,003, 30,020 and 31,691 in the FCG, MCG, FTG and MTG, respectively. The results demonstrated that TTX administration resulted in a decreased number of piRNAs in females, while an increased number of piRNAs in males. The total unique piRNA is 65,535 (Table 1 and Table S1), including known 943 piRNAs, about 1.439% of the total unique piRNAs. 98 female-specific and 90 male-specific piRNAs (Table S1). There were 60,497 unique piRNAs that display less than 10 reads in all groups (Table S1). A total of 79 piRNA clusters were isolated based on the characteristics of the piRNA clusters (Table S2). These piRNA clusters exist and are unevenly distributed on each chromosome (Table S2). Two PIWI protein-coding genes, *piwil1* and *piwil2*, were identified from transcriptome data in *Takifugu flavidus*. The transcriptome analysis revealed that *piwil1* and *piwil2* were both expressed in the ovary and testis, and the expression level in the testis is relatively higher than that in the ovary. Among them, the expression level of *piwil1* was always higher than *piwil2* in both ovaries and testes. After TTX administration, both *piwil1* and *piwil2* were down-regulated in the testis, whereas *piwil1* was up-regulated in the ovary with no significant difference (Table S4). These results indicated that *piwil1* and *piwil2* were more sensitive to TTX in the testis than the ovary of *Takifugu flavidus.*

### Transcriptome analysis of differentially expressed genes and piRNAs between the FCG and MCG

**Fig. 2:**
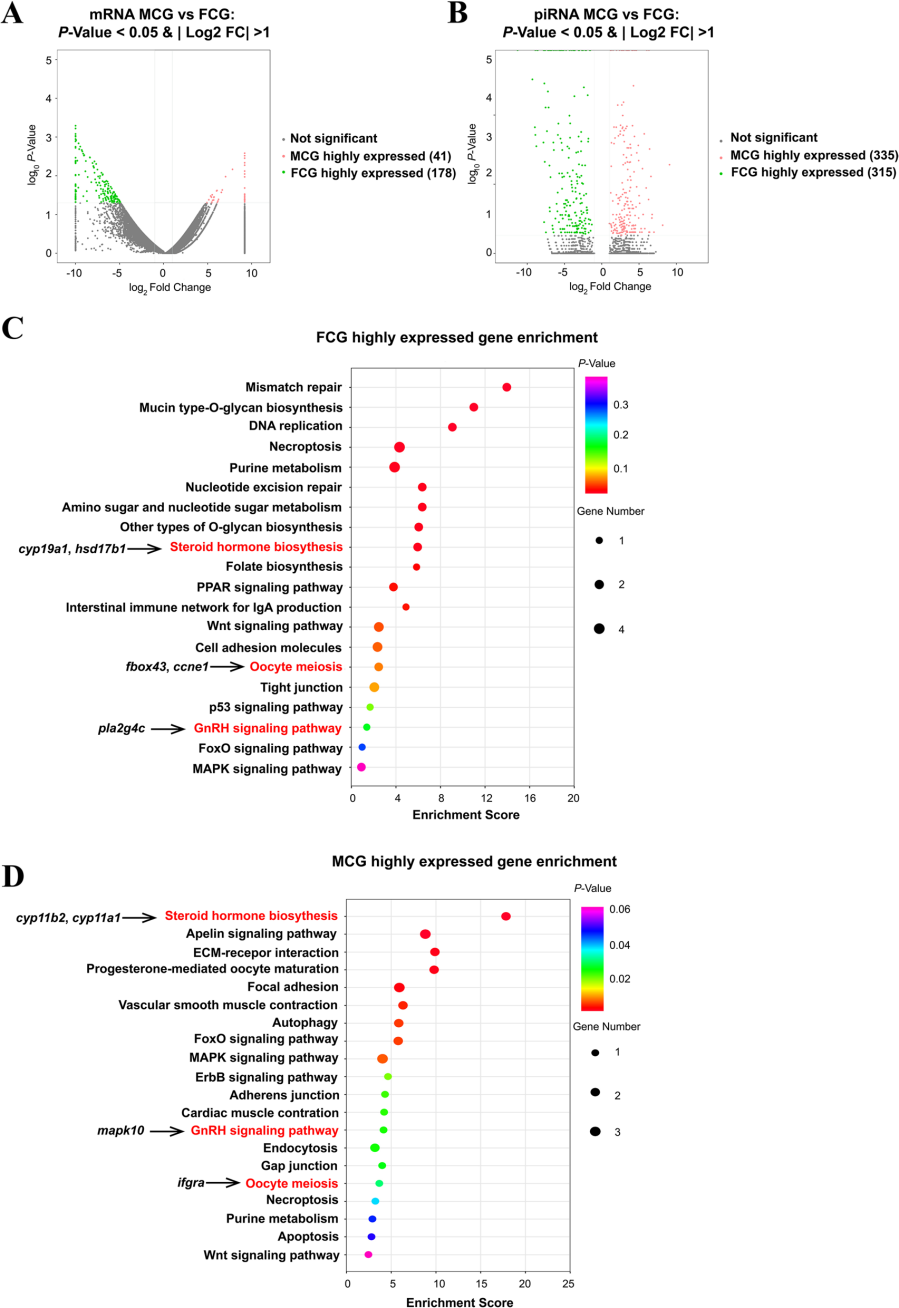
The differential expression of piRNAs and mRNAs between MCG and FCG. **A**-**B**, Volcano plot showing the differential expression genes (**A**) or piRNAs (**B**) in MCG vs FCG, the red dot represents up-regulated genes/piRNAs, the green dot represents down-regulated genes/piRNAs, the gray dot represents no significant difference; **B**, KEGG analysis enrichment pathway of FCG highly expressed genes in the ovary; **C**, KEGG analysis enrichment pathway of MCG highly expressed genes in the testis

Overall distribution of differentially expressed mRNAs and piRNAs was observed on the volcano plot. By comparing MCG and FCG, a total of 219 differentially expressed genes were identified with *P* < 0.05 and |log2 (fold change) |> 1 using the base mean method, and 660 differentially expressed piRNAs were identified with *P* < 0.05 and |log2 (fold change) |> 1 using the TPM method. 41 genes and 335 piRNAs were differentially expressed in MCG, 178 genes and 315 piRNAs were differentially expressed in FCG (Fig. 2A-B). The female dominant genes such as *cyp19a1* (cytochrome P450 19A1-like) and *foxl2* (Forkhead Box L2) were identified in the FCG, and the male dominant genes such as LOC101075863/*cyp11b2* (cytochrome P450 11B, mitochondrial) and *dmrt1* (doublesex and mab-3 related transcription factor 1) were identified in the MCG (Table S4).

KEGG (Kyoto Encyclopaedia of Genes and Genomes) pathway enrichment analysis of FCG and MCG differentially expressed genes showed that steroid hormone biosynthesis, oocyte meiosis and GnRH signaling pathway were both enriched in MCG and FCG. However, the involved genes are different, such as *cyp19a1* and *hsd17b1* (hydroxysteroid 17-beta dehydrogenase 1) enriched in FCG’s, *cyp11b2* and *cyp11a1/LOC101063139/* (cholesterol side-chain cleavage enzyme, mitochondrial) enriched in MCG’s steroid hormone biosynthesis. Folate biosynthesis, p53 signaling and some DNA replication/repair were found to be specifically enriched in FCG. Apoptosis and autophagy were found to be specifically enriched in MCG (Fig. 2C-D).

### Transcriptome analysis of up and down regulated genes and piRNAs between the FCG and FTG

**Fig. 3:**
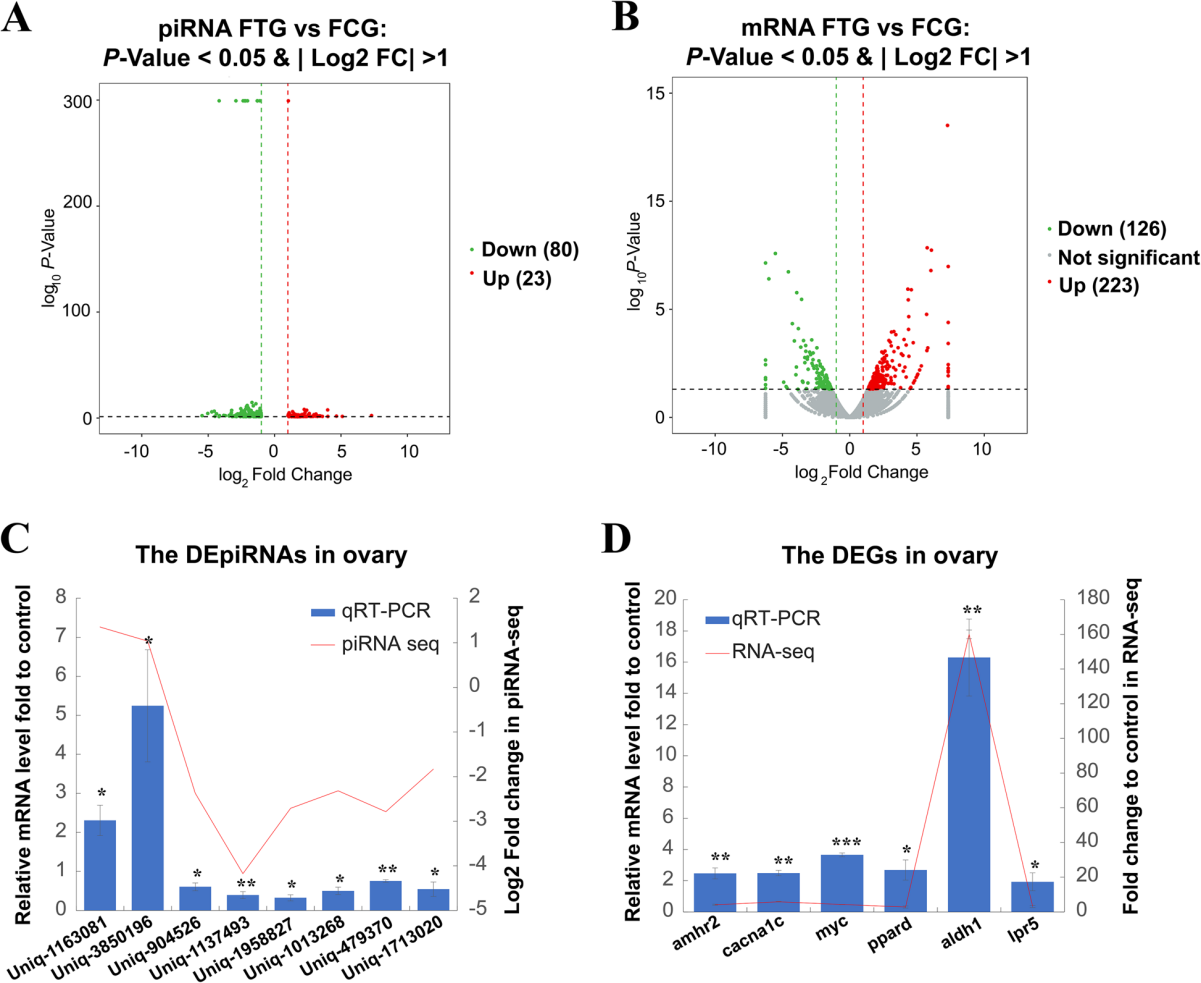
The differential expression of piRNAs and mRNAs in the FTG and FCG. **A**-**B**, Volcano plot showing the differential expression piRNAs (**A**) or genes (**B**) in FTG vs FCG, the red dot represents up-regulated genes/piRNAs, the green dot represents down-regulated genes/piRNAs, the gray dot represents no significant difference; **C**, Relationship between relative expression levels of selected piRNA in the FTG and FCG validated by qRT-PCR and log2 fold changes derived using piRNA sequencing, FCG as a calibrator; **D**, qRT-PCR and mRNA sequencing of selected genes between FTG and FCG, FCG as a calibrator. Expression levels of piRNA/mRNA measured with qRT-PCR were depicted with column charts, and the red line represents the log2 fold changes/fold changes in sequencing. Error bars represent one standard deviation of three different biological replicates. The internal reference gene is *U6* for piRNA and *gapdh* for mRNA. Set as * *P* < 0.05, ** *P* < 0.01 and *** *P* < 0.001 vs controls. The asterisks represent significant differences between the treatment group and the control group. Statical significance is reported for each piRNA or mRNA

By comparing FTG and FCG, TTX administration resulted in 23 piRNAs and 223 genes expression up-regulated, meanwhile, 80 piRNAs and 126 genes expression down-regulated in the ovaries (Fig. 3A-B and Table S2, S4).

To investigate the reliability of differentially expressed genes and piRNAs in transcriptomes. Eight relative highly expressed piRNAs (*uniq_1163081*, *uniq_3850196*, *uniq_556841*, *uniq_1137493*, *uniq_1958827*, *uniq_1013268*, *uniq_479370* and *uniq_1713020*) and six genes (*amhr2*, anti-Mullerian hormone receptor type 2; *cacna1c*, calcium voltage-gated channel subunit alpha1 C; *myc*, MYC proto-oncogene bHLH transcription factor a; *ppard*, peroxisome proliferator activated receptor delta; *LOC101078894/adh1*, alcohol dehydrogenase 1-like and *LOC101069468/lpr5*, low-density lipoprotein receptor-related protein 5-like) were screened for further verification by qRT-PCR (Quantitative Real-Time PCR). The piRNAs and genes’ expression changes of trend between the FTG and FCG in the qRT-PCR results were almost consistent with the transcriptome data, these results indicating that the transcriptome data was reliable (Fig. 3C-D and Table S1, S4).

**Fig. 4:**
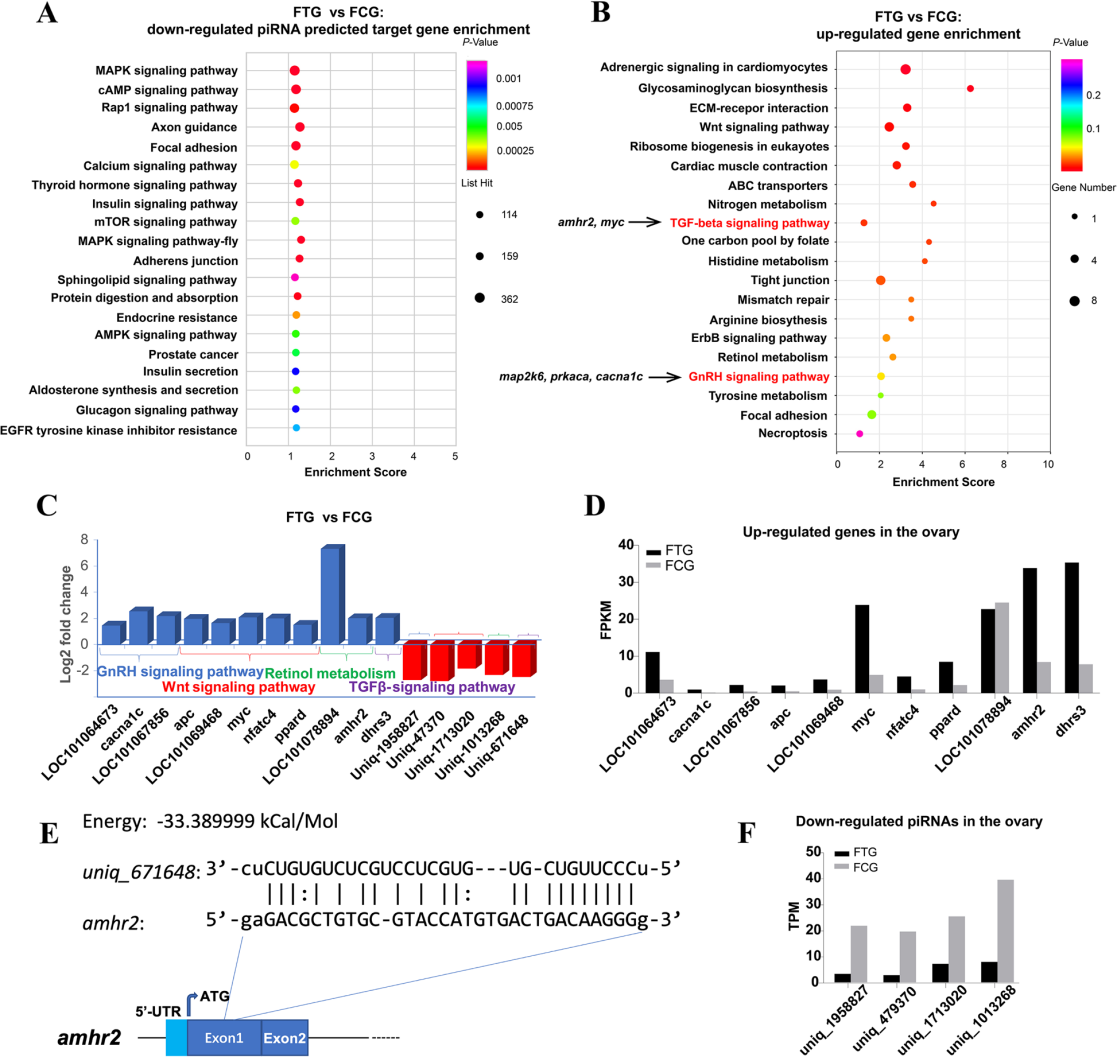
Transcriptome analysis of up and down regulated genes and piRNAs between the FCG and FTG. **A**, KEGG analysis enrichment pathway of the down-regulated piRNA predicted target genes in the FTG vs FCG; **B**, KEGG analysis enrichment pathway of the up-regulated genes in the FTG vs FCG; **C**, The relative expression of genes and piRNA in the FTG vs FCG based on piRNA and mRNA sequencing, open brace in the same colour indicated the same signaling pathway; **D**, The expression value of FPKM (mRNA, upper part) and TPM (piRNA, lower part) of selected genes/piRNAs in FTG vs FCG; **E**, The *uniq_671648*-target RNA (*amhr2*) pairing prediction by MiRnada. The light blue box showed the 5’-UTR sequence and the navy blue box showed the exons of the target gene

KEGG and GO (Gene Ontology, which was shown in Table S7-8) pathway enrichment analysis of FTG compared FCG down-regulated piRNA target genes showed that these genes mainly enriched in signaling pathways, such as MAPK, cAMP, Rap 1, calcium, thyroid hormone, insulin, and AMPK signaling pathway (Fig. 4A and Table S7-8). KEGG pathway enrichment analysis of FTG compared FCG up-regulated genes showed that these genes mainly enriched in metabolism pathways, such as nitrogen, histidine, retinol and tyrosine metabolism. Furthermore, *amhr2*, and *myc* were enriched in the TGF*β* signaling pathway, *LOC101064673*/*map2k6* (dual specificity mitogen-activated protein kinase kinase 6), *LOC101067856*/*prkaca* (cAMP-dependent protein kinase catalytic subunit alpha), *cacna1c* were enriched in GnRH signaling pathway (Fig. 4B and Table S5-6).

To further confirm the correlation between down-regulated piRNAs and up-regulated expression genes, KEGG pathway enrichment analysis of FTG compared FCG down-regulated piRNA and up-regulated genes were compared. Up-regulated genes enriched in the oocyte meiosis, progesterone-mediated oocyte maturation, and steroid hormone biosynthesis were selected for mapping with the down-regulated piRNAs in FTG. The results showed that *uniq_1958827* predicated target gene *cacna1c*; *uniq_1013268* predicated target gene *LOC101078894*/*adh1*; *uniq_479370* predicated target gene *LOC101069468/lpr5*; *uniq_1713020* predicated target gene *ppard*; *uniq_671648* predicated target gene *amhr2* were identified. *cacna1c* was involved in the GnRH signaling pathway, *adh1* was involved in retinol metabolism; both *lpr5* and *ppard* were involved in the Wnt signaling pathway; and *amhr2* was involved in the TGF*β* signaling pathway (Fig. 4C and Table S5, S7). These genes/piRNAs' relative expression values in FTG and FCG were shown using RPKM/TPM (Fig. 4D). In addition, the *uniq_671648*-*amhr2* mRNA pairing structure was shown in Fig. 4E.

### Transcriptome analysis revealed gene expression feminization of testis after TTX administration

**Fig. 5:**
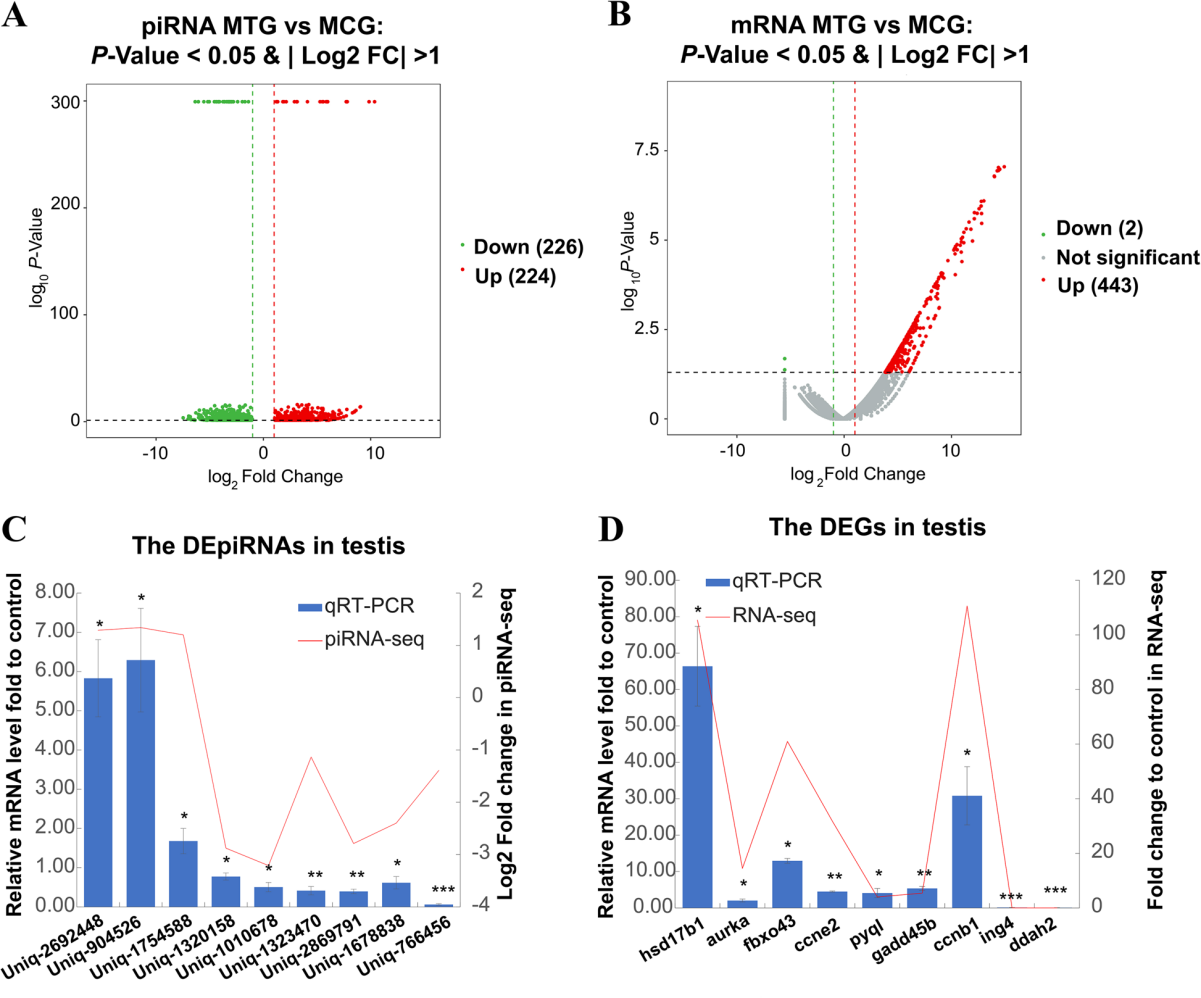
The differential expression of piRNAs and mRNA in the MTG and MCG. **A**-**B**, Volcano plot showing the differential expression piRNAs (**A**) or genes (**B**) in MTG vs MCG, the red dot represents up-regulated genes/piRNAs, the green dot represents down-regulated genes/piRNAs, the gray dot represents no significant difference; **C**, Relationship between relative expression levels of selected piRNA in the MTG and MCG validated by qRT-PCR and log2 fold changes derived using piRNA sequencing, MCG as a calibrator; **D**, qRT-PCR and mRNA sequencing of selected genes between MTG and MCG, MCG as a calibrator. Expression levels of piRNA/mRNA measured with qRT-PCR were depicted with column charts, and the red line represents the log2 fold changes/fold changes in sequencing. Error bars represent one standard deviation of three different biological replicates. The internal reference gene is *U6* for piRNA and *gapdh* for mRNA. Set as * *P* < 0.05, ** *P* < 0.01 and *** *P* < 0.001 vs controls. The asterisks represent significant differences between the treatment group and the control group. Statical significance is reported for each piRNA or mRNA

By comparing MTG and MCG, TTX administration resulted in 224 piRNAs and 443 genes expression up-regulated, meanwhile, 286 piRNAs and 2 genes (*ddah2*, dimethylarginine dimethylaminohydrolase 2 and *ing4*, an inhibitor of growth family member 4) expression down-regulated in the testes, the female dominant genes such as *cyp19a1*, *gdf9* (growth differentiation factor 9) and *foxl2* were identified up-regulated in the MTG (Fig. 5A-B and Table S1, S4).

To investigate the reliability of differentially expressed genes and piRNAs in transcriptomes, nine relative highly expressed piRNAs (*uniq_2692448*, *uniq_904526*, *uniq_1754588*, *uniq_1320158, uniq_1010678*, *uniq_1323470*, *uniq_2869791*, *uniq_1678838* and *uniq_766456*), the up-regulated genes including *hsd17b1*, *ccnb1* (cyclin B1)*, aurka* (aurora kinase A), *fbxo43* (F-box protein 43), *ccne2/LOC101065671* (cyclin N-terminal domain-containing protein 2-like), *pygl* (glycogen phosphorylase L), *gadd45β/LOC101080011* (growth arrest and DNA damage-inducible protein GADD45 beta) and the down-regulated two genes *ing4* and *ddah2* in the MTG were screened for further verification by qRT-PCR. The qRT-PCR results confirmed a good correlation with transcriptome sequencing results in piRNA and gene expression level (Fig. 5C-D and Table S1, S4), these results also indicated that the transcriptome data was reliable.

**Fig. 6:**
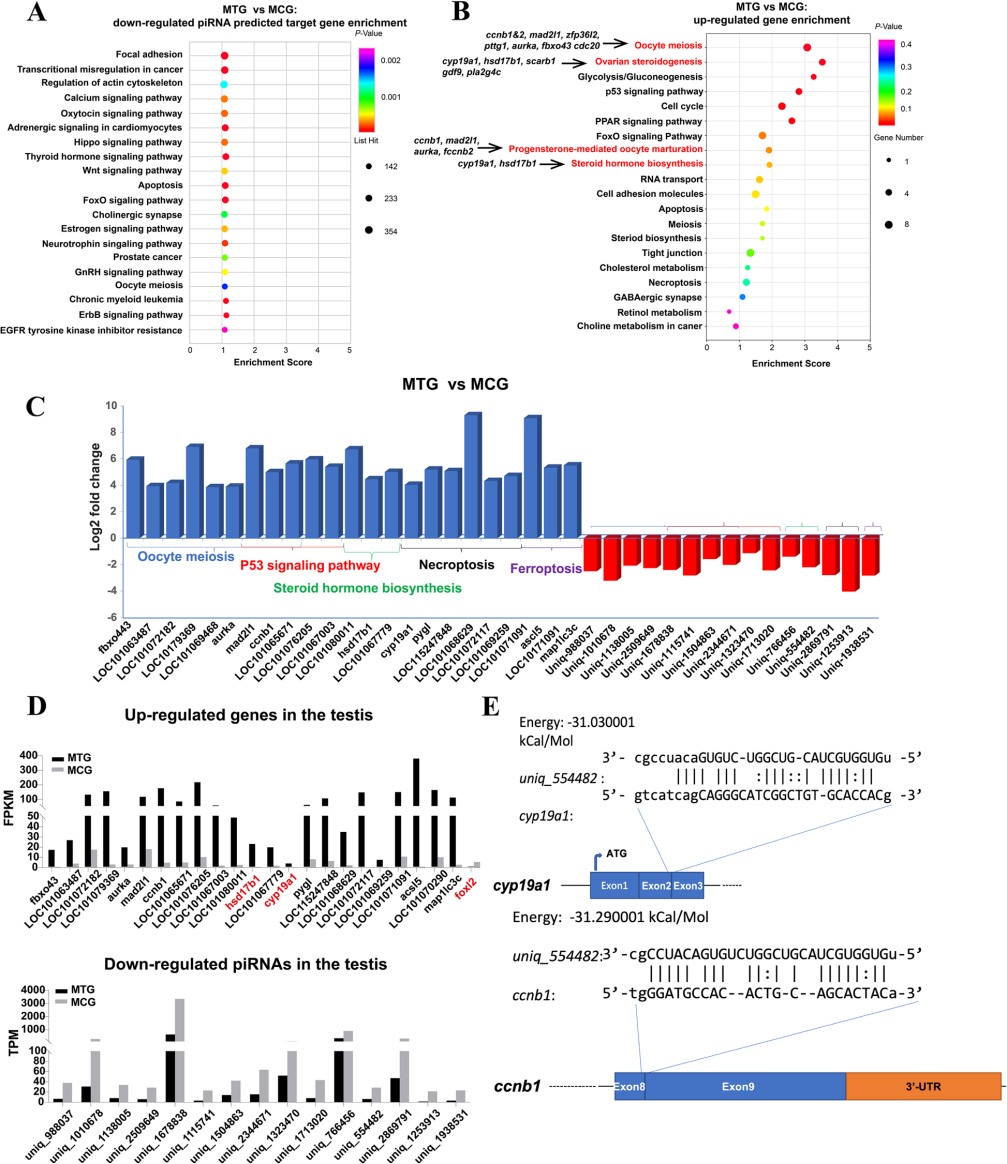
Transcriptome analysis of up and down regulated genes and piRNAs between the MCG and MTG. **A**, KEGG analysis enrichment pathway of the down-regulated piRNA predicted target genes in the MTG vs MCG; **B**, KEGG analysis enrichment pathway of the up-regulated genes in the MTG vs MCG; **C**, The relative expression of genes and piRNA in the MTG vs MCG based on piRNA and mRNA sequencing, open brace in the same color indicated the same signaling pathway; **D**, The expression value of FPKM (mRNA, upper part) and TPM (piRNA, lower part) of selected genes/piRNAs in MTG vs MCG; **E**, *uniq_554482*-target RNA (*cyp19a1* and *ccnb1*) pairing prediction by MiRnada. The yellow box showed the 3’-UTR sequence and the navy blue box showed the exons of the target gene

KEGG pathway enrichment analysis of MTG compared MCG down-regulated piRNA target genes showed that these genes also mainly enriched in signaling pathways, such as calcium, oxytocin, adrenergic, Hippo, thyroid hormone, wnt, foxO, estrogen, neurotrophin, GnRH and erbB signaling pathway (Fig. 6A and Table S7-8). KEGG pathway enrichment analysis of MTG compared MCG up regulated genes showed that these genes mainly enriched in ovary differentiated related pathways, such as ovarian steroidogenesis and steroid hormone biosynthesis, oocyte meiosis and progesterone-mediated oocyte maturation; the cell apoptosis-related pathways, such as cell cycle, tight junction, PPAR and p53 signaling pathway (Fig. 6B). Furthermore, *ccnb1*, *ccnb2/LOC101076205* (G2/mitotic-specific cyclin-B2-like), *cdc20/LOC101063487* (cell division cycle 20), *mad2l1*, *zfp36l2/LOC101079369* (ZFP36 ring finger protein-like 2), *pttg1/LOC101072182* (PTTG1 regulator of sister chromatid separation, securin), *aurka*, *fbxo43* were enriched in oocyte meiosis; *cyp19a1*, *hsd17b1*, *scarb1* (scavenger receptor class B member 1), *gdf9*, *pla2g4c/LOC101069259* (cytosolic phospholipase A2-gamma) were enriched in ovarian steroidogenesis; *ccnb1*, *ccnb2*, *mad2l1* and *aurka* were enriched in progesterone-mediated oocyte maturation; *cyp19a1*, *hsd17b1*, *hsd17b2/LOC101067779* (very-long-chain 3-oxoacyl-CoA reductase-A) were enriched in Steroid hormone biosynthesis (Fig. 6B and Table S5-6). These results indicated the gene expression feminization in testis after exogenous TTX administration.

To further confirm the correlation between down-regulated piRNA and up-regulated expression genes, KEGG pathway enrichment analysis of MTG compared MCG down-regulated piRNA and up-regulated genes were compared. Up-regulated genes enriched in oocyte meiosis, progesterone-mediated oocyte maturation, steroid hormone biosynthesis, p53 signaling pathway, ferroptosis, necroptosis and cellular senescence were selected for mapping with the down-regulated piRNAs in MTG. The results showed that *uniq_1010678* predicated target gene *ccne2/LOC101065671*; *uniq_1138005* predicated target gene *ccnb2*; *uniq_1678838* predicated target gene *aurka*; *uniq_2509649* and *uniq_1115741* predicated target genes *mad2l1*; *uniq_1504863* and *uniq_2344671* predicated target gene *ccnb1*; *uniq_988037* predicated target gene *cdc20*; *uniq_1323470* predicated target gene *fbxo43*; *uniq_766456* predicated target gene *hsd17b1*; *uniq_554482* predicated target gene *cyp19a1*; *uniq_1713020* predicated target gene *gadd45β*; *uniq_2869791* and *uniq_1253913* predicated target gene *pygl* and *uniq_1938531* predicated target gene acyl-CoA synthetase long chain family member 5 (*acsl5*) were identified (Fig. 6C and Table S3). The genes *ccnb1*, *mad2l1* (mitotic arrest deficient 2 like 1), *zfp36l2/LOC101079369*, *pttg1/ LOC101072182*, *aurka*, *fbxo43*, *ccnb2/ LOC101076205* and *cdc20/LOC101063487* were involved in the oocyte meiosis or progesterone-mediated oocyte maturation pathway, *hsd17b1* and *cyp19a1* were involved in the steroid hormone biosynthesis, the gene *gadd45β/LOC10108001* was involved in the p53 signaling pathway, *pygl* was involved in necroptosis and *acsl5* was involved in ferroptosis (Fig. 6C). These genes/piRNAs’ relative expression values in MTG and MCG were shown using RPKM/TPM (Fig. 6D). In addition, the *uniq_554482-cyp19a1*/*ccnb1* RNA pairing structures were shown in Fig. 6E.

### Transcriptome analysis of sexual dimorphism distribution mechanism of TTX in gonads

**Fig. 7:**
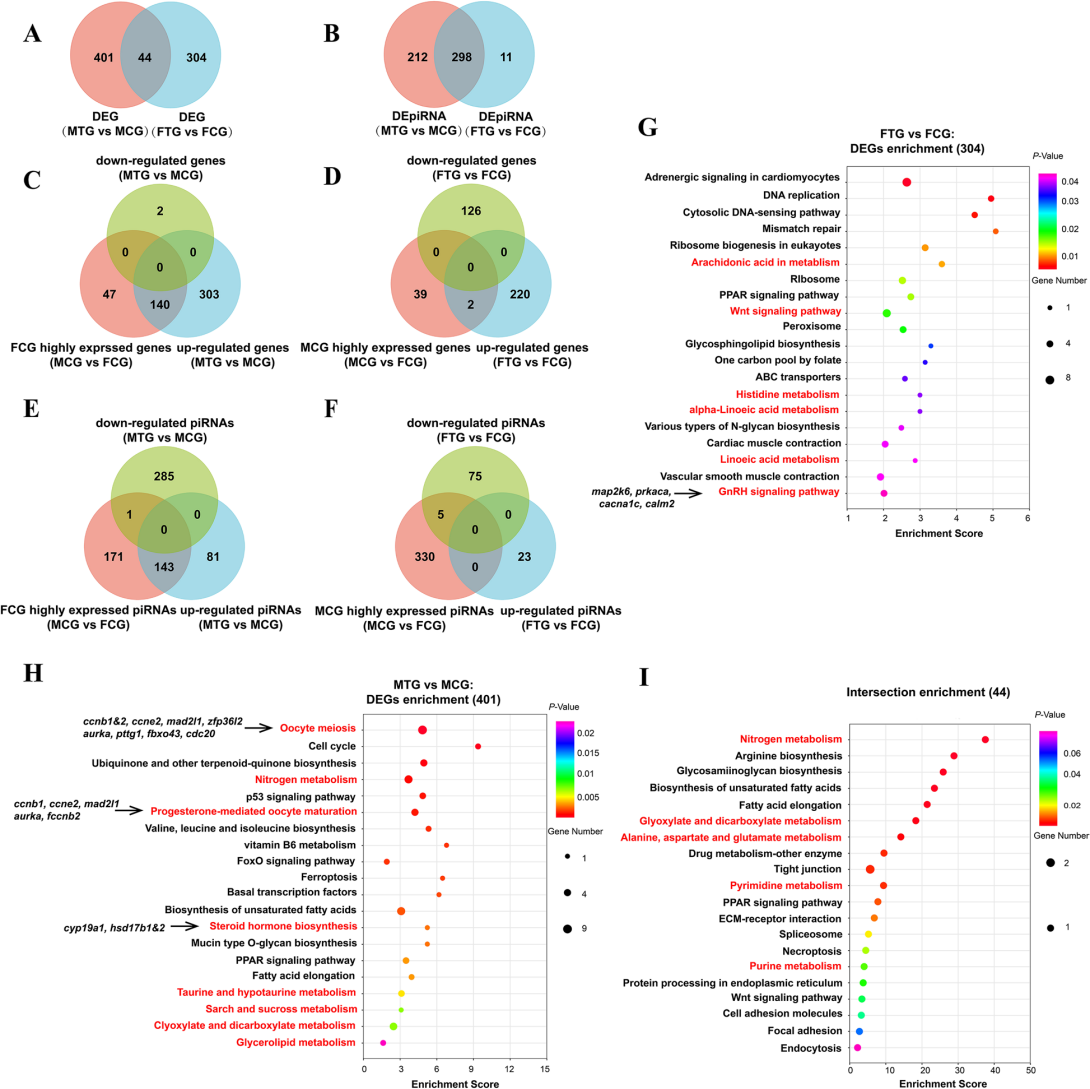
Transcriptome analysis of sexual dimorphism distribution mechanism of TTX in gonads. **A**-**B**, Venn plot showing the differentially expressed genes (**A**) or piRNAs (**B**) in the DEGs/DEpiRNAs (FTG vs FCG) vs DEG/DEpiRNA (MTG vs MCG); **C**-**D**, Venn plot showing the differentially expressed genes (**C**) or piRNAs (**D**) in the pairwise FCG highly expressed genes (MCG vs FCG) vs up-regulated gene (MTG vs MCG) vs down-regulated gene (MTG vs MCG); **E**–**F**, Venn plot showing the differentially expressed genes (**E**) or piRNAs (**F**) in the pairwise MCG highly expressed genes (MCG vs FCG) vs up-regulated gene (FTG vs FCG) vs down-regulated gene (FTG vs FCG); **G**, KEGG analysis enrichment pathway of of 44 common DEGs shared by DEGs (MTG vs MCG) vs DEGs (FTG vs FCG); H, KEGG analysis enrichment pathway of 304 DEGs (FTG vs FCG); **I**, KEGG analysis enrichment pathway of 401 DEGs (MTG vs MCG)

To gain insight into the sexual dimorphism distribution mechanism of TTX in the ovary and testis. Differentially expressed genes (DEGs) and piRNAs (DEpiRNAs) in MTG vs MCG and FTG vs FCG were compared analysis. Venn diagram analysis showed that 401 and 304 DEGs were found to be significantly differentially expressed in MTG vs MCG and FTG vs FCG groups, respectively. And 44 DEGs were common between these two groups (Fig. 7A). There were 212 and 11 DEpiRNAs significantly differentially expressed in MTG vs MCG and FTG vs FCG groups, respectively. And 298 DEpiRNAs were common between these two groups (Fig. 7B).

Common and differentially expressed DEGs/DEpiRNAs among six groups, including FCG and MCG highly expressed genes/piRNAs groups, FTG vs FCG and MTG vs MCG up-regulated genes/piRNA groups, and FTG vs FCG and MTG vs MCG down-regulated genes/piRNA groups, were compared analyzed. As the Venn diagram showed that 47, 2 and 303 differentially expressed genes were found in FCG highly expressed genes, MTG vs MCG down- and up-regulated genes groups, the FCG highly expressed genes group shared 140 common genes with MTG vs MCG up-regulated genes group (Fig. 7C). The same pattern is also seen in the piRNA comparison group (Fig. 7E). In contrast, 39, 126 and 220 differentially expressed genes were found in MCG highly expressed genes, FTG vs FCG down and up-regulated genes groups, the MCG highly expressed genes group only shared 2 common genes with FTG vs FCG up-regulated genes group (Fig. 7D). The same pattern was also seen in the piRNA comparison group (Fig. 7F). These results further indicated that exogenous TTX can cause feminization of testis.

In the KEGG enrichment analysis of differentially expressed DEGs in FTG vs FCG and MTG vs MCG groups (304 and 401 genes), the results showed that compared with FTG vs FCG group, differentially expressed DEGs in MTG vs MCG group were more enriched in metabolism pathway, including nitrogen, vitamin B6, taurine and hypotaurine, starch and sucrose, glyoxylate and dicarboxylate and glycerolipid metabolism pathways (Fig. 7G-H). KEGG pathway enrichment analysis of 44 shared common DEGs of MTG vs MCG and FTG vs FCG, the results showed that they were also mainly enriched in metabolism pathways, including nitrogen, glyoxylate and dicarboxylate, alanine, aspartate and glutamate, drug-other enzymes, pyrimidine and purine metabolism pathways (Fig. 7I). These results suggested that the effect of TTX on gonads was direct. Compared with the ovary, the testis produced more metabolic pathways in response to exogenous TTX, which might be a reason for the sexual dimorphism of TTX distribution in gonads.

### Exogenous TTX induces cell apoptosis in testis

**Fig. 8:**
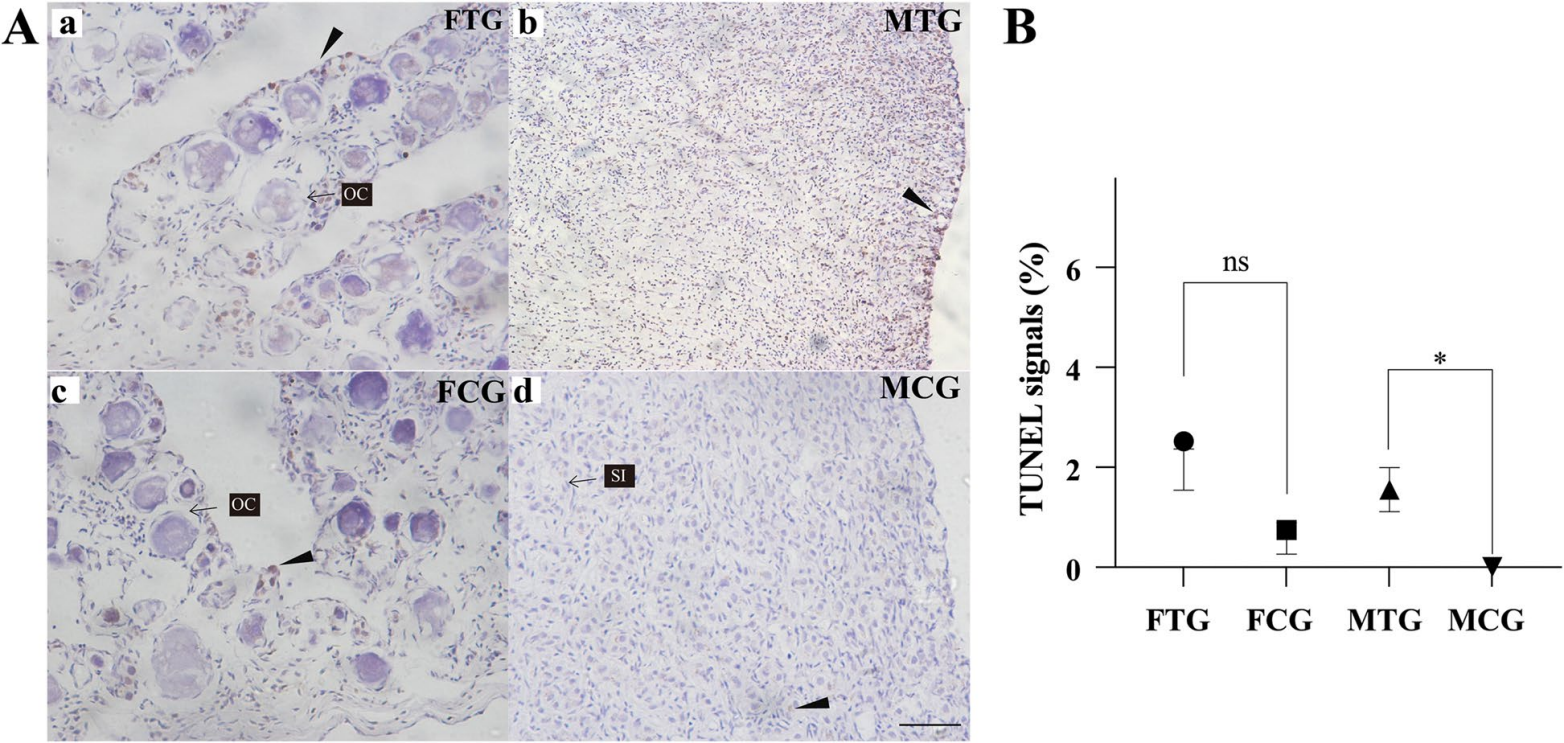
Exogenous TTX induces cell apoptosis in the testis of *Takifugu flavidus*. **A**, TUNEL staining of *Takifugu flavidus* gonads after TTX administration. a and c, the ovary of FTG and FCG; b, d, testis of MTG and MCG; The dark brown signals are apoptosis signals by TUNEL staining and the black triangle arrows are indicating the positive signals, the scale bar is 50 μm. **B**, Apoptosis ratio of gonads in juvenile *Takifugu flavidus* after TTX administration*.* Data were shown as mean ± SEM, *, indicating that *P* < 0.05

The piRNA and mRNA transcriptome analysis showed that cell apoptosis-related genes were up-regulated in testis and related piRNA were down-regulated. To further confirm the damaging effects of TTX on the testis, TUNEL assay (TdT-mediated dUTP-biotin nick end labeling) was used for identifying apoptotic signals or cells in situ. Immunohistochemical analysis showed that positive signals were detected in the somatic cells nuclei of the FTG, FCG ovary and MTG testis. Little to no signal of apoptosis was observed in the MCG testis (Fig. 8A). Statistical analysis of the area of the positive signals, the results showed no significant differences between FTG and FCG, whereas, the positive signals in MTG were significantly higher than that in the MCG (Fig. 8B). These results indicated that exogenous TTX administration induces cell apoptosis in testis.

## Discussion

### Sex differentiated *Takifugu flavids* showed sexual dimorphism on response to exogenous TTX

Processes involving sex determination and reproduction were complicated and regulated by various internal and/or external factors, particularly in pufferfish which showed a sexual dimorphism of TTX accumulation in gonads. The molecular data available was limited for *Takifugu* to know about the exact internal mechanism or role of TTX in the development of gonads. The accumulation and tissue-specific distribution of TTX in pufferfish, mainly in the genus *Takifugu* have been widely investigated from the viewpoint of the TTX-resistance VGSCs expression and distribution [2, 10, 47]. Evidence also showed that TTX is usually significantly at a high level during the maturation/spawning period than in the ordinary period [1, 4, 5, 9, 17]. The present study also demonstrated that the TTX concentration level is quite low in 6-month-old *Takifugu flavids*, and after TTX administration, TG level of TTX was significantly higher than CG in kidney, cholecyst, skin, liver, heart, muscle, ovary and testis, which was reflected by LC–MS/MS and fluorescence immunohistochemistry analysis, these results consistent with the exogenous TTX administration in *Takifugu rubripes *[48–50].

The artificially cultured 6-month-old *Takifugu flavids* were chosen for the first time to investigate the piRNAs and mRNAs expression changes in separate sex of pufferfish. Firstly, from a seasonal perspective, whole body TTX level was low in this period, accumulation of TTX also demonstrated sex dimorphism, ovary accumulated a higher level of TTX than the testis. Meanwhile, “Endogenous” TTX was also found in the ovary at this stage, TTX immunofluorescence signals were detected indicating the TTX was also accumulated in the ovary before the exogenous TTX treatment. At this time, endogenous TTX levels in the gonads do not reach the high levels compared with sexual maturation period, which can avoid the potentially serious or to be ignored effects of exogenous TTX administration. Secondly, TTX in the developing stage of pufferfish, especially for the gonads marginally been investigated. Also, at this time, *Takifugu flavids* has been sex differentiated, and it was reflected by clear histological differences, i.e., the ovary has an ovarian cavity and oocytes have initiated meiosis and formed growing oocytes, the testis only available the spermatogonia without initiated meiosis. Thirdly, after exogenous TTX administration, the TTX amount of both the testis and ovary was significantly increased. Meanwhile, the transcriptome data and analysis also showed more piRNAs and genes in the testis were affected by TTX than that in ovaries. Therefore, the response to exogenous TTX in this period is sufficient and worthy of in-depth study.

### piRNAs play a role in the regulation of sex related genes’ expression after TTX administration

Previous studies demonstrated that piRNAs are indispensable for generating functional germ cells, gametogenesis, and female infertility [22, 33, 51]. and often bound to members of the piwi protein family to realize its regulation function [22, 24, 27, 28, 52]. Two PIWI proteins, *piwil1* and *piwil2*, were identified in *Takifugu flavidus*, the results were consistent with another pufferfish *Takifugu fasciatu*s, which also reported having two Piwils that were abundantly expressed in the immature gonads [53]. *piwil1* and *piwil2* were both expressed in the ovaries and testes, and the expression level in the testis is relatively higher than that in the ovary of *Takifugu flavids*. After TTX administration, both *piwil1* and *piwil2* were down-regulated in the testis, whereas *piwil1* was up-regulated in the ovary with no significant difference. The present study predicted piRNA *uniq_1504863*, *uniq_1302131* and *uniq_2344671* might be bound to these two Piwi proteins, and these results indicated that *piwil1* and *piwil2* were more sensitive to TTX in the testis in *Takifugu flavidus*.

Interestingly, up-regulated genes in the MTG were enriched in steroid hormone biosynthesis and ovarian steroidogenesis pathways, and the related female dominant gene *cyp19a1* was identified. A piRNA *uniq_554482* was also screened in the down-regulated piRNAs in MTG, and the *uniq_554482* predicted the target gene including *cyp19a1*. In addition, another ovarian factor *gdf9* and steroidogenesis related gene *hsd17b1* were also significantly up regulated after TTX administration. The down-regulated piRNAs *uniq_2093102* (targeted *gdf9*) and *uniq_766456* (targeted *hsd17b1*) were also identified and involved in the upregulated genes in the MTG, showing the impact of TTX on genes’ expression may be through the piRNAs in the testis, and indicated that piRNAs play a role in the regulation of sex related genes after TTX administration.

### Ovaries are more tolerant to exogenous TTX than testis

The present study revealed that ovaries are more tolerant to exogenous TTX than testis, this is reflected by a smaller number of genes and piRNAs that were changed after TTX administration and not significantly elevated TUNEL positive signals in the ovary. In addition, the total number of piRNAs is greatly increased in the testis but slightly decreased in the ovary after exogenous TTX administration. These results indicated that TTX has a bigger influence on the testis. There are two possible reasons for this phenomenon. First, the ovary itself has the accumulation of TTX and was more tolerant to exogenous TTX, so there is little change in piRNA quantity. The second is exogenous TTX administration can lead to feminization of testicular gene expressions, such as up-regulated expression of female-dominant genes. We speculate that the increased piRNAs in the testis silence more transposons or genes to resist/reduce the toxicity of TTX.

As previous studies reported that the p53 signaling pathway is involved in the regulation of cell cycle arrest, senescence and apoptosis [54–56], necroptosis and ferroptosis pathways were also involved in the regulation of cell death [56–58]. Transcriptome analysis in this study indicated that the apoptosis, p53 signaling and necroptosis pathways were found in MTG vs MCG up-regulated genes group in testis, whereas only the necroptosis pathway was found in FTG vs FCG up-regulated genes group in the ovary. These results were consistent with the TUNEL analysis of the present study that exogenous TTX induced more cell apoptosis in the testis than the ovary.

Furthermore, it’s hard to observe the changes in somatic and germ cells from the view of histological or HE results after 8 h of TTX administration. In general, the changes of nucleic acids are always earlier than protein and histological changes, for example, tilapia had formed primitive gonads 5 days after hatching, and there was no difference at the histological level between XX and XY individuals at this period [59]. However, at the molecular level, XX individuals had begun to express Cyp19a1a, while XY individuals did not. In contrast, XY individuals had begun to express Amh (anti-Müllerian hormone) and Dmrt1, while XX individuals did not [59].

### Testis demonstrated feminized gene expression patterns and up-regulated metabolic pathways after TTX administration

The molecular mechanism of sexual dimorphism in TTX accumulation in gonads of pufferfish is largely unknown, the relationship between TTX accumulation and sexual steroidogenesis, sex differentiation related genes also needs to be clarified. In terms of piRNA and mRNA levels, the present study found that exogenous TTX administration would feminize gene expression in the testis, female-dominant genes *foxl2*, *cyp19a1/ cyp19a1a* and *gdf9* were up regulated in the MTG. The c*yp19a1* is the key gene encoding cytochrome P450 aromatase, which is responsible for estrogen production in the ovary [60–62], the increase of *cyp19a1a* expression might lead to an increased level of endogenous estrogen E2 (17beta-estrodiol), excessive estrogen also induces the initiation of cell apoptotic pathways by Stabilizing Schlafen-12 Protein Turnover [63], leading to a significant increase in cell apoptosis in the testis [63]. Estrogen is also considered a key factor in sex differentiation [64, 65]. It is well documented that *foxl2* and *cyp19a1* play a vital role in promoting ovarian development and maintaining feminization afterward in teleosts [38, 63, 64, 66, 67]. Growth differentiation factor 9 (*gdf9*) is a member of the TGF*β* superfamily. As an oocyte-derived growth factor, *gdf9* plays key role in regulating follicle development [68, 69]. Steroidogenesis is critical for gamete maturation in the teleost, more essentially oogenesis [70], except *cyp19a1*, *hsd17b1* was found to be up-regulated in the MTG. Moreover, KEGG analysis of differentially expressed genes/piRNAs in MTG also enriched in Wnt signaling and oocyte meiosis pathway. Studies have shown that the components of the Wnt signaling pathway are responsible for reproductive functions, including the regulation of follicular maturation and the production of steroids in the ovaries [71–74]. The B-type cyclins take important roles in oocyte meiosis [75], the *ccnb1* and *ccnb2* are essential for the is critically required for the proliferation of gonocytes [76], and both were upregulated by the TTX. These up-regulated key female dominant genes and pathways induced feminization of testicular gene expression, which may need to mimic the female endocrine environment to counter the damaging effects of TTX on the testis.

The TGF*β* signaling pathway regulates growth, division and proliferation in the ovary [77, 78], one of the TGF*β* signaling pathway member *amhr2* were up-regulated after TTX administration FTG, the *amhr2* was associated with sex determination in *Takifugu rubripes*, and showed regulation of germ cell proliferation [77–80]. Combined with the differential analysis base on piRNA sequencing, the up-regulated gene involved in the down-regulated piRNA in the ovary, indicated TTX may impact the gene expression of the ovary through the regulation of piRNA and showed a positive effect.

The present study compared differentially expressed piRNAs and genes in MTG vs MCG and FTG vs FCG, these two groups shared 298 common piRNAs and 44 common genes with the changes. KEGG pathway enrichment analysis of these 44 common genes showed that TTX directly affects piRNAs and mRNAs changes by influencing nucleotide, lipid, glycan, carbohydrate and protein metabolism as well as cell genetic information processes and signals. On the other hand, compared with the ovary, the testis produced more metabolic pathways in response to exogenous TTX. KEGG pathway enrichment analysis of 401 DEGs significantly differentially expressed in MTG vs MCG group, indicating the testis have richer metabolic and/or transport pathways than ovary to against the accumulation of TTX. These results indicated that TTX plays a detrimental role in testicular development, the testes themselves may be repellent to TTX.

Above all, our research revealed that the response of ovary and testis to TTX administration was largely different, ovary are more tolerant to TTX (has fewer piRNA and mRNA expression changes), whereas testes are more senstive to TTX. Exogenous TTX administration mainly resulted in testis gene expression pattern's feminization, up regulation of more metabolic pathways and increased apoptosis. These data will deepen our understanding on the accumulation of TTX sexual dimorphism in *Takifugu*.

## Conclusion

In conclusion, the present research highlights the differentially expressed piRNAs and genes after TTX administration in gonads. The response of ovary and testis to TTX administration was largely different, ovary are more tolerant to TTX, whereas testes are more sensitive to TTX. The differentially expressed piRNAs potential regulated genes and the differentially expressed gene were partly overlapping. The regulatory role of TTX may through piRNA on genes related to reproduction support the notion that piRNAs play crucial roles in reproduction and the TTX novel function in pufferfish reproduction. Exogenous TTX administration mainly resulted in testis gene expression pattern's feminization, up regulation of more metabolic pathways and increased apoptosis in *Takifugu rubripes*. Further confirmed that excessive TTX is harmful to testicular development. Overall, this research will deepen our understanding on the accumulation of TTX sexual dimorphism in *Takifugu*.

## Materials and methods

### Animals and chemicals

The experimental animals, artificial cultured marine pufferfish *Takifugu flavidus* juveniles (6-month-old, 100.0 ± 10.0 g body weight, 100 individuals) were purchased from Shanghai Aquatics Research Institution. Crystalline of TTX (Supelco, USA) was used in the administration experiment and as a standard sample for the liquid-chromatography-tandem mass spectrometry (LC–MS/MS) analysis. All other chemicals were reagent grade (Sinopharm, China). The present study on *Takifugu flavidus* was carried out following the relevant guidelines and regulations (following the ARRIVE guidelines [81]). The protocols were approved by the academic ethics committee of Shanghai Ocean University.

### TTX administration and sample preparation

The administration experiment was performed at the laboratory of the Shanghai Ocean University. The TTX was directly dissolved in 0.1% acetic acid to 1 mg/ml, then diluted with 0.7% saline solution to 0.25 μg/μl. The test fish received an intramuscular administration of 0.25 μg TTX/g body weight into the caudal muscle as described previously and were maintained in an 80-L oxygenated plastic tank of aerated artificial seawater at 20 ℃ [48]. After 8 h of administration, twelve fish of control and TTX-treated group were randomly collected, followed ice-cold anesthetized and dissected. The gonads were dissected immediately and separated into two parts, 1/4 gonad was fixed in 4% PFA for histological and immunofluorescence histochemical observation; another 3/4 of gonad were stored at -80 ℃ until RNA extraction. Kidney, cholecyst, skin, liver, heart, and muscle were also dissected and stored at -20 ℃ until RNA exaction.

### TTX extraction and quantification

Tissues (kidney, cholecyst, skin, liver, heart, and muscles) were extracted with 0.1 M HCl as reported previously [48, 82]. The extracts from the tissues were defatted with ethoxyethane and centrifuged at 12,000 rpm for 10 min. The resulting supernatants were ultrafiltered (Amicon Ultra, Millipore, USA), then submitted to LC–MS/MS (Liquid Chromatography with tandem mass spectrometry) for TTX determination with some modification [83, 84]. In the LC–MS/MS analysis, chromatography was carried out using a Xevo TQ-XS system (Waters, Milford, USA) with an XBridge Amide column (2.1 mm × 100 mm, particle size 1.7 μm,) and mobile phase comprising 45% 0.1 mM methanoic acid and 55% acetonitrile at a flow rate of 350 μL/min. The eluate was introduced into a MassLynxTM N detector (Waters, Milford, USA) in which the TTX was ionized by positive-mode electrospray ionization with a desolvation temperature of 550 °C, the fragment ion at m/z 162 that results from the dissociation of the parent ion of TTX at m/z 320 was detected by the multiple reaction monitoring modes. These tissue extracts were spiked with TTX standard [83, 85, 86].

### Histological observation and TTX immunofluorescence histochemistry

For histological observation and immunofluorescence histochemical analyses, the *Takifugu flavidus* gonads were dissected and fixed in 4% PFA (Paraformaldehyde) for 12 h at 4℃, then dehydrated, embedded in paraffin, and sectioned at 5 μm. Hematoxylin–eosin staining and immunofluorescence histochemical staining were performed as described previously [87, 88]. The antibody against TTX was purchased from CD (Creative diagnostics, USA) and was diluted at 1:200, Hoechst 33,258 (Sigma, USA) was diluted at 1:1000 staining the nucleic acid. A Nikon ECLIPES 80i light microscope (Nikon, Japan) was used to image the H&E (hematoxylin–eosin staining) stained sections. Leica TCS SP8 X (Leica, German) confocal microscope was used to image fluorescence-stained sections. The TTX signals ratio of gonads was statistical analysis of the three different areas of each sample using ImageJ (NIH, USA) and Graph Prism 8.0 (San Diego, USA).

### TUNEL analysis

TdT-mediated dUTP-biotin nick end labeling (TUNEL) technique was applied to evaluate the apoptotic response of TTX administration in the ovary and testis. Reactions were performed on Sects. (5 µm) of 4% Paraformaldehyde (PFA)-fixed (overnight at 4 °C) and paraffin-embedded ovaries and testes. Apoptotic cells with DNA breaks were detected using Colorimetric TUNEL Apoptosis Assay Kits (#C1091, Beyotime Institute of Biotechnology, Shanghai, China). TUNEL assay was then performed according to the instructions by the manufacturer. Images were used a Nikon ECLIPES 80i light microscope (Nikon, Japan). The apoptosis signal ratio of gonads was a statistical analysis of the three different areas of each sample under TUNEL staining by using ImageJ (NIH, USA) and Graph Prism8.0 (San Diego, USA) as described before.

### Transcriptome sequencing

After sex is determined by histological observation, 3 or 4 gonad samples are mixed into one pool for each FTG, MTG, FCG and MCG group. Total RNAs of each group were extracted using RNAiso Plus (Takara, Japan) [89]. The agarose electrophoresis was used to detect the integrity of total RNA, and the Agilent 2100 Bioanalyzer (Agilent Technologies, USA) was used to detect the concentration and purity of total RNA. Then, four cDNA libraries were constructed following the manufacturer’s recommendations and sequenced on the Illumina HiSeq platform, generating 150-bp paired-end reads, a summary of the raw and clean data were shown in Table 1. High-quality clean reads from each sample were mapped onto the assembled transcript sequences using Bowtie software (Version 2.2.2) [90] with default parameters. The RSEM program [91] was then used to estimate the expression abundance of the transcripts. The total number of mapped reads for each transcript was determined and then normalized to determine FPKM (Fragments Per Kilobase Million).

For piRNA, four small RNA libraries were constructed using TruSeq Small RNA Donor Prep Kits (Illumina, USA). The small RNA sequencing analysis was performed on the Illumina Hiseq 2500 sequencing platform by the Novegene Company (Beijing, China). The small RNA with 15–41 nt were selected after screening the sequence of Q-score > 20, removing the N base, and filtering out the inappropriate lengths. The clean reads were classified and annotated by comparing them with relevant databases using Bowtie and BLAST [90]. The small RNAs were classified by filtering clean reads using the Rfam database [92] as well as aligning with the genome of *fTakRub1.2* genome (closely related species *Takifugu rubripes*, fTakRub1.2, E-value < 0.01, Bioproject number PRJEB31988/PRJNA543527, Assembly under accession number GCA_901000725.2 at NCBI, National Center for Biotechnology Information, https://www.ncbi.nlm.nih.gov). Sequences without aligned transcripts were matched with the miRBase database by zero-base mismatch [93, 94], and the miRNA in the sequence was filtered as far as possible. Then, the remaining sequences with 26–32 nt unannotated reads analyzed by Piano were predicted as novel piRNAs [95] based on using the structure and sequence features of transposon-piRNAs interactions, the novel piRNA aligned with piRBase (http://bigdata.ibp.ac.cn/piRBase/) using bowtie by zero-base mismatch [96], the aligned sequences were considered as known piRNAs. The genome was used to match with the clean sequences for piRNA to determine various small RNAs. Annotated non-coding RNAs included rRNAs, tRNAs, snRNA, miRNA, piRNA, etc. These aligned RNAs were subjected to BLAST against Rfamv.10.1 (http://www.sanger.ac.uk/software/Rfam) [92], GenBank (http://www.ncbi.nlm.nih.gov/genbank/) databases and miRBase v.21 database (http://www.mirbase.org/) [94]. The known piRNAs were identified by aligning against the piRBase release v2.0 database (http://www.regulatoryrna.org/database/piRNA/) [96], and the expression patterns of known piRNAs and novel piRNA in four groups were analyzed.

### Bioinformatic analysis and data statistics

For mRNA, DESeq software standardized the counts’ number of each group gene and calculated the fold of difference and used NB (negative binomial distribution test) to test the significance of the difference in the number of reads [97]. The expression level of genes can be calculated using FPKM (Fragments Per Kilobase Million). The formula is FPKM = (total exons reads)/ (mapped reads, millions × exon length, kb) [98]. Finally, the differential protein-coding genes were screened according to the different folds and the different significance test results. DEGs were identified with a threshold of *P* < 0.05 and |log2 (fold change) |> 1 based on the BaseMean method [97]. Due to no biological replicates in this study, the *P* value was calculated with the DEG algorithm [99] in the R package including the Audic-Claverie statistic as above [100]. The differential transcripts/genes were classified according to the official annotation of the Gene Ontology (GO) [101] and Kyoto Encyclopedia of Genes and Genomes (KEGG) [32, 102–104] was analyzed.

Differentially expressed piRNAs (DEpiRNAs) were identified with a threshold of *P* < 0.05 and |log2 (fold change) |> 1. Due to no biological replicates in this study, the *P* value was calculated with the DEG algorithm [99] in the R package including the Audic-Claverie statistic [100]. In addition, a quantitative analysis of piRNAs was performed. The expression level of piRNAs is directly reflected by their abundance. The higher the abundance of piRNAs is, the higher the expression level is. In piRNA sequencing analysis, the expression level of piRNAs can be calculated by locating the piRNA sequence of *Takifugu flavidus* using the transcripts per million (TPM). The formula is TPM = (number of reads aligned for each piRNA) / (sample Total aligned reads) × 10^6^ [105]. Differential expression analysis aims to identify differentially expressed piRNAs among different groups based on the TPM method [97]. After obtaining differential expression, all the expressed piRNA sequences in four groups are compared with the human genome using sRNAmapper software (version 1.0.5) [106], the parameters are default, the obtained map file, and then use proTRAC software (version 2.4.2) to do piRNA cluster prediction [107]. Then the target genes of differentially expressed piRNAs were predicted using the MiRanda software with a score of 150 or higher and free energy -30 or lower [108].

The enrichment analysis of the Gene Ontology (GO) function and Kyoto Encyclopedia of Genes and Genomes (KEGG) pathway was performed as previously reported [32, 101–104]. The false discovery rate (FDR) was used to adjust the *P* value and GO or KEGG terms (FDR < 0.01) were considered significantly enriched.

### qRT-PCR validation

Gonadal piRNA and mRNA expression were also assayed using qRT-PCR. Total RNA was extracted following the above description. cDNAs were synthesized using HiScript® II 1st Strand cDNA Synthesis Kit (Vazyme, China). Hieff UNICON® Universal Blue qPCR SYBR Green Master Mix (Yeason, China) was used for quantitative qRT-PCR assays. The gene names and the primers are listed in Table S9. *Gadph* was used as an internal control [89, 109, 110]. The relative abundances of mRNA transcripts were evaluated using the formula: R = 2^−ΔΔCt^ [111].

For piRNA, 1 μg of total RNA above as a template for synthesis of the first-strand cDNA with a Mir-X miRNA First-Strand Synthesis (Takara, Japan) following the manufacturer’s instructions. The expression of the piRNAs used a miScript SYBR Green PCR Kit (Takara, Japan) and followed the manufacturer’s instructions on a CFX96 Real-Time PCR System (Bio-rad, USA). The primers for piRNA qRT-PCR were listed in Table S9. We used *U6* as an internal reference gene to control for differences among samples. Each group sample of four or three mixed fishes was run triple times and the relative quantification of piRNA expression was calculated using the 2^−ΔΔCt^ method [111].

mRNA and piRNA expression levels of each sample were normalized to the levels of *gadph* and *U6* gene in the same samples, respectively. the control group was set as 1 and was omitted in the figures, the mRNA and piRNA level folds to control were shown in figures. This method was reported in previous research [112].

### Statistical analysis

Except where otherwise indicated, Statistical analyses were performed in the GraphPad Prism 9.0 (San Diego, California USA) environment. Each measurement was repeated at least three times. data are presented as mean ± SEM, and the *P* values were determined by two-tailed Student’s t-tests. *P* < 0.05 was considered to be statistically significant.

## Supplementary Information

**Additional file 1: Table S1.** Relative expression of all piRNAs identified and DEpiRNAs in gonads.

**Additional file 2: Table S2.** Prediction of piRNA clusters.

**Additional file 3: Table S3.** List of preidicted target genes of DEpiRNAs.

**Additional file 4: Table S4.** Relative expression of mRNAs and DEGs in gonads.

**Additional file 5: Table S5.** KEGG analysis of DEGs in gonads.

**Additional file 6: Table S6.** GO analysis of DEGs in gonads

**Additional file 7: Table S7.** DEpiRNA target gene KEGG analysis.

**Additional file 8: Table S8.** GO analysis of DEpiRNA target genes.

**Additional file 9: Table S9.** Primers and for RT-qPCR.

**Additional file 10: Figure s1.** Length range and first base bias of piRNA in the gonad of juvenile *Takifugu flavidus*.

## Data Availability

All sequencing raw data have been deposited in the Sequence Read Archive (SRA) database under Bioproject number PRJNA769942 (https://www.ncbi.nlm.nih.gov/bioproject/PRJNA769942). the *Takifugu rubripes* reference genome *fTakRub1.2* under Bioproject number PRJEB31988/PRJNA543527, Assembly under accession number GCA_901000725.2 at NCBI, National Center for Biotechnology Information, https://www.ncbi.nlm.nih.gov).
