# Supplementary material for: Transcriptome analysis revealed gene expression feminization of testis after exogenous tetrodotoxin administration in pufferfish Takifugu flavidus
Source: BMC Genomics. 2022 Aug 3;23:553. doi: 10.1186/s12864-022-08787-z (PMC9347094; doi:10.1186/s12864-022-08787-z)
Supplement: Supplementary file 10 — Additional file 10: Figure s1. Length range and first base bias of piRNA in the gonad of juvenile Takifugu flavidus. [file 12864_2022_8787_MOESM10_ESM.docx]

**Figure s1**


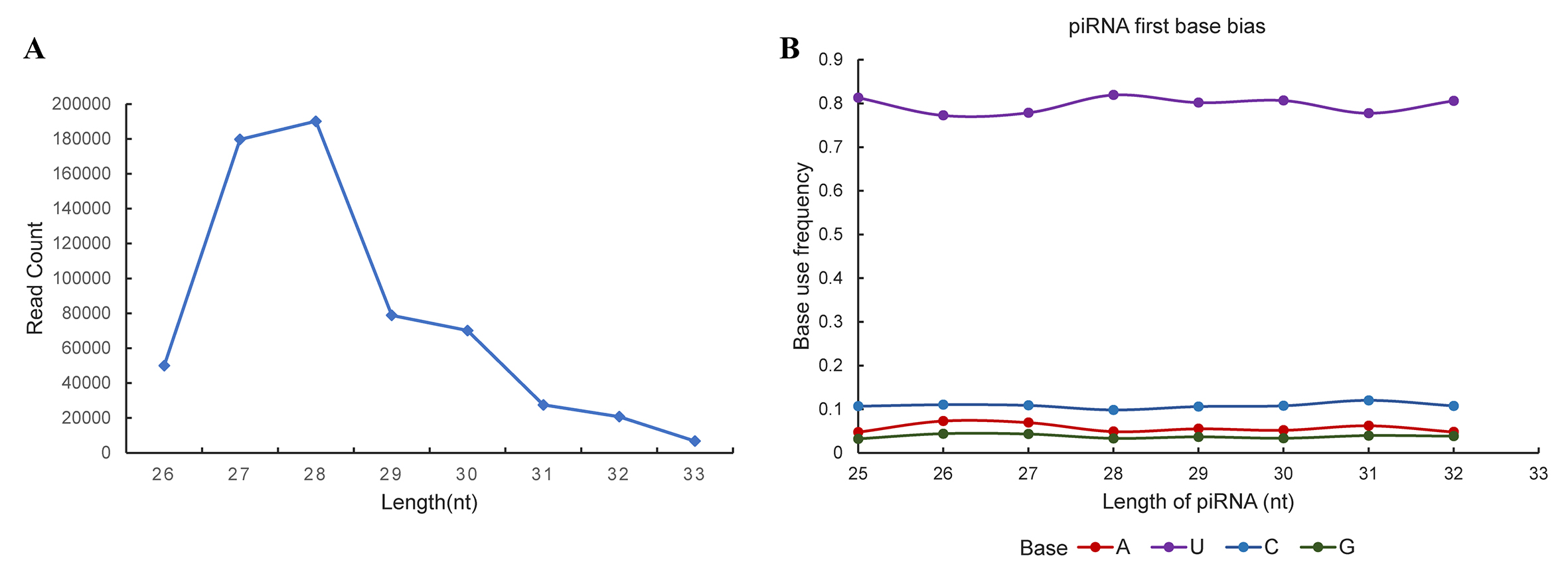


**Figure s1** Length range and first base bias of piRNA in the gonad of juvenile *Takifugu flavidus*. A length distribution of sequenced piRNA from TTX-treated and control gonads of juvenile *Takifugu* *flavidus*. The X-axis denotes the piRNA length of 26-31 nucleotides, Y-axis represents the read count of piRNA; B, the first base bias of the sequenced piRNA from TTX-treated and control gonads of juvenile *Takifugu flavidus*. The X-axis denotes the piRNA length of 26-31 nucleotides, Y-axis represents the percentage of the base use frequency.
